# Turning Wastes into Resources: Red Grape Pomace-Enriched Biscuits with Potential Health-Promoting Properties

**DOI:** 10.3390/foods13142195

**Published:** 2024-07-11

**Authors:** Annalisa Giosuè, Francesco Siano, Luigia Di Stasio, Gianluca Picariello, Chiara Medoro, Marta Cianciabella, Rosalba Giacco, Stefano Predieri, Ermanno Vasca, Olga Vaccaro, Rosaria Cozzolino

**Affiliations:** 1 Nutrition, Diabetes and Metabolism Unit, Department of Clinical Medicine and Surgery, “Federico II” University, Via S. Pansini 5, 80131 Naples, Italy; rosalba.giacco@isa.cnr.it (R.G.); olga.vaccaro@unina.it (O.V.); 2 Department of Pharmacy, “Federico II” University, Via D. Montesano 49, 80131 Naples, Italy; 3 Institute of Food Sciences (ISA), National Research Council, Via Roma 64, 83100 Avellino, Italy; luigia.distasio@isa.cnr.it (L.D.S.); picariello@isa.cnr.it (G.P.); rosaria.cozzolino@isa.cnr.it (R.C.); 4 Institute for Bioeconomy (IBE), Italian National Research Council, Via P. Gobetti 101, 40129 Bologna, Italy; chiara.medoro@ibe.cnr.it (C.M.); marta.cianciabella@ibe.cnr.it (M.C.); stefano.predieri@ibe.cnr.it (S.P.); 5 Department of Chemistry and Biology “A. Zambelli”, University of Salerno, Via Giovanni Paolo II 232, 84084 Fisciano, Italy; evasca@unisa.it

**Keywords:** red grape pomace, enriched biscuits, cardiometabolic health, in vitro simulated digestion, antioxidant fibers, anthocyanins, volatile organic compounds

## Abstract

The life-long adherence to a dietary pattern able to provide a high amount of polyphenols demonstrating beneficial cardiometabolic effects is demanding for the general population. In this study, red grape pomace (GP) was used as an ingredient to increase the daily polyphenols’ intake. The incorporation of crude crushed GP at 20 and 30% (*w*/*w*) in a control (CTR) biscuit formula improved the nutritional profile by increasing the fiber and reducing lipid and energy content while providing up to 540 mg_GAE_ of polyphenols per 100 g. Besides anthocyanins, GP contains flavonoids and grape-seed procyanidins, contributing to the remarkable antioxidant capacity of 20- and 30-GP biscuits. Upon in vitro gastro-duodenal enzymatic digestion, the concentration of reducing sugars for 20-GP and 30-GP compared to the CTR biscuits dropped significantly, meaning that the combined action of GP fibers and polyphenols could delay the intestinal absorption of glucose. Overall, 60 volatiles were detected in biscuits. All in all, the content of Maillard reaction products was lower in GP than in CTR biscuits, possibly due to the free radical scavenging ability of polyphenols. Despite the high rates of GP utilized, the sensorial attributes and the overall liking of the GP biscuits—especially the 20-GP ones—were not substantially affected. These findings will support nutritional studies to assess the potential role as functional foods of GP biscuits, and, afterwards, the large-scale production of a food mainly based on a waste ingredient turned into a resource.

## 1. Introduction

Biscuits represent a staple food item in the diet of most populations worldwide. According to the latest available statistics for Europe, Italy has the second highest per capita volume of biscuit consumption, with 10.5 kg per person per year, after the United Kingdom at 13.6 kg [1].

By and large, biscuits are consumed on a daily basis, being suitable for many eating occasions, from breakfast to after dinner snacking. They can come in large “family-size” boxes or in single-serving packages, easy to take along, all highly available in the current food environment, where they can also be more affordable than other (often healthier) products. Indeed, despite their widespread diffusion, biscuits are generally characterized by a suboptimal nutritional profile, being a source of high Glycemic Index (GI) carbohydrates, sugar and saturated and/or *trans* fatty acids. Additionally, sweet biscuits can contribute, though marginally, to the maximum recommended daily salt intake, despite being among those food products people do not consider to be salty [2].

In recent years, functional nutrition has become increasingly popular and more consumers are inclined to make purchasing decisions driven by the positive health effects achievable consuming foods with enhanced functionality [3]. Within this scenario, besides the careful selection of raw materials (e.g., organic, unrefined, low-gluten, etc.), the use of functional ingredients has a pivotal role. Since fruit and vegetable by-products are packed with high-value compounds [4], their use in the frame of a circular economy has the potential to meet not only the “functional” requirements for human health, but also the advocated need of the environmental sustainability of food production. A variety of ingredients share these features, but grape pomace (GP) is among the most studied and used in the preparation of functional foods. GP is the major by-product of wine, consisting of grape skins, pulps and seeds, all being a valuable source of bioactive compounds, in particular polyphenols, fiber and unsaturated fatty acids. Depending on the conditions of the grapes when they are harvested, the residues may represent from 13.5% to 14.5% of the total volume of grapes [5], posing a pressure for their management at the industrial level that can be attenuated by redirecting this underutilized coproduct from the winemaking to the food industry. Phenolic compounds in GP have indeed the potential for improving cardiometabolic health [6], as phenolic GP extracts have been specifically reported to exert antioxidant, anti-inflammatory and anti-hyperglycemic effects [7,8]. Thus far, epidemiological evidence has clearly indicated that the habitual consumption of polyphenol-rich foods (e.g., fruit, vegetables, whole grains, etc.) is associated with a lower risk of cardiovascular disease incidence [9], and interventions in humans with diets naturally rich in polyphenols have demonstrated their ability to reduce the postprandial blood glucose response [10], to improve fasting and postprandial dyslipidemias and to reduce oxidative stress [11]. However, the life-long adherence to a dietary pattern able to provide the high amount of polyphenols that has demonstrated beneficial cardiometabolic effects (around 3 g day^−1^) [10] is demanding for people who are not diagnosed with a disease but who aim to improve their habitual diet in the perspective of long-term cardiometabolic prevention. Therefore, the use of functional foods with concentrated amounts of polyphenols could be a suitable solution to fill the “polyphenol gap” between the intake ensured by the habitual diet, and the one needed to achieve beneficial effects on health. This strategy is supported by one previous study from our group, which showed that the consumption of a functional drink rich in polyphenols extracted from red grape pomace is able to improve insulin sensitivity in young healthy individuals [7].

To date, many studies have reported the incorporation of GP in a variety of food products, from fruit jam to drinks, pasta and bakery products, including cookies [12,13,14]. Indeed, dried GP can be crushed or pulverized to obtain whole GP flour, which is a suitable alternative flour to replace conventional ones in the making of products showed to be convincing for their chemical, technological and sensory characteristics [15]. In particular, the use of crushed whole GP in biscuits can be a *win-win* strategy to ameliorate the nutritional profile of a product that is largely consumed but made of ingredients (i.e., sugar, refined flour, saturated fats) with potential harmful effects on cardiometabolic health, and, at the same time, to contribute to the daily polyphenol intake through a dietary tool associated with pleasure. Among all bakery products, biscuits enriched with GP flour showed, indeed, the best global acceptance by consumers [15]. However, to the best of our knowledge, the percentage of substitution of conventional flours for GP flour was substantially limited to 5 to 20%. Our aims were to use whole crushed red GP at high incorporation rates to develop 100% plant-based biscuit prototypes, and to determine their chemical and nutritional composition with an extended approach, together with the evaluation of sensorial properties and overall liking.

Our final aim is to evaluate if the GP biscuit prototypes characterized can be used as dietary tools to increase the polyphenol intake within the daily diet of the general population.

## 2. Materials and Methods

### 2.1. Biscuit Production

#### 2.1.1. Raw Materials

Red GP resulting from winemaking of Aglianico Irpino, which was composed of skin and seeds, was donated by Feudi San Gregorio spa winery (Sorbo Serpico, AV, Italy). The crude GP flour was obtained by crushing dried GP using a regular food processor. The other ingredients (i.e., soft wheat flour, sugar, baking powder, soy drink) were purchased at an organic food market (EcorNaturaSì Spa, Bologna Italy). The extra-virgin olive oil was produced at the Oleificio Sociale Cooperativo of Alberobello, Puglia, Italy.

#### 2.1.2. Biscuit Formulas

**Figure 1:**
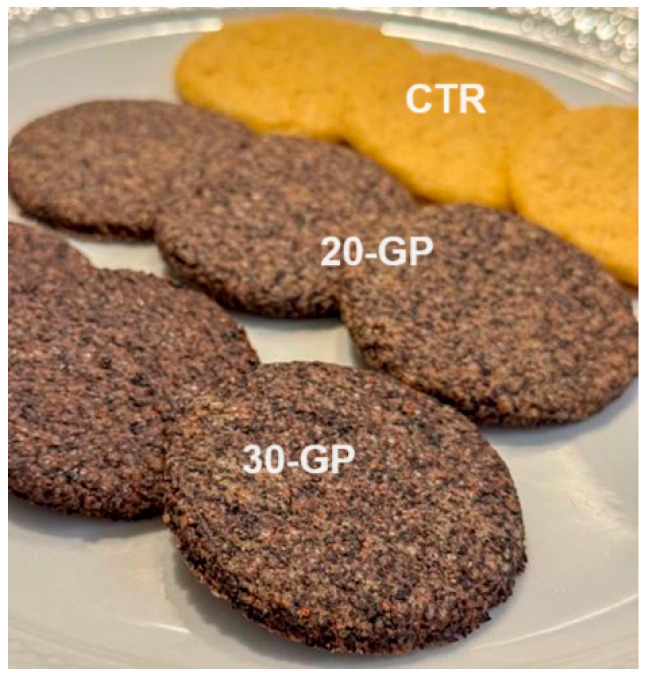
Biscuit prototypes with 30% (30-GP), 20% (20-GP) of grape pomace and control (CTR) biscuits after baking.

Biscuits were produced from a base formula (control biscuit, CTR) containing organic wheat flour (40 g 100 g^−1^), white sugar (20 g 100 g^−1^), extra-virgin olive oil (20 g 100 g^−1^), organic soy drink with no added sugars (15 g 100 g^−1^) and organic baking powder (5 g 100 g^−1^). The total phenolic compounds of olive oil was 21 mg 100 g^−1^, as determined with the Folin–Ciocalteu assay (see below). Whole crushed GP was used at the amount of 20 g 100 g^−1^ in the 20-GP biscuits and at the amount of 30 g 100 g^−1^ in the 30-GP biscuit formula. As a result, in the two formulas, crushed whole GP replaced the 20% or the 30% (*w*/*w*) of each ingredient included in the base formula (Supplemental Table S1). These two percentages were selected as a compromise to maximize the health potential benefits while ensuring the dough workability. Biscuits were formed and patterned to 6 cm diameter and 0.3 cm thickness, and baked in the oven at 130 °C for 25 min (Figure 1).

### 2.2. Product Characterization

#### 2.2.1. Proximate Composition and Energy Content

The physicochemical composition (moisture, crude protein, total lipids, ash) of GP and biscuit prototypes was determined using the Official Methods of Analysis of AOAC International. The total crude protein of samples was determined through the Kjeldahl method using 6.25 as the nitrogen conversion factor [16]. Lipids were extracted from seeds and flour with the Soxhlet method and stored at −20 °C until further analysis. The moisture content was determined gravimetrically in triplicate after heating 5.0 g of the sample placed on Pirex glass dishes in an oven at 105 °C for 6 h up to constant weight. For the analysis of ashes, 5.0 g of the sample was accurately weighted and incinerated in a muffle furnace at 550 °C until gray ashes and constant weight were obtained. After cooling, ash was weighted, and percent content determined either on a dry basis or on a wet basis considering moisture. Total carbohydrates were determined by the difference in percentages: 100% − (crude protein + total lipids + ash + moisture)%. The total dietary fiber in GP and biscuit samples was determined by the AOAC method 985.29 [17].

The energy content of crude GP and biscuit prototypes was calculated by multiplying the grams of protein, lipid, available carbohydrates and fiber content in 100 g of each product per 4, 9, 3.75 and 2, respectively, and adding the resulting energies from each macronutrient to obtain the kilocalories per 100 g of product.

#### 2.2.2. Preparation of FAME and GC-FID Analysis of Fatty Acids

Analysis of the fatty acid methyl esters (FAMEs) was performed according to the AOAC method 969.33 [18]. FAMEs were obtained through trans-methylation, which was performed by suspending 200 mg of the Soxhlet-extracted lipid fraction in 2 mL of 1.25 M HCl/CH3OH solution in Pyrex test tubes with screw caps and incubated in a water bath at 90 °C for 60 min. After the addition of 2 mL of deionized water, FAMEs were extracted with *n*-hexane and filtered using Millex 0.45 µm PVDF disposable syringe filters (EDM Millipore Corp., Billerica, MA, USA). GC analyses were performed using a 7890A gas chromatograph (Agilent Technologies, Palo Alto, CA, USA) equipped with a flame ionization detector (FID), using an SP-2380, 100 m × 0.20 mm capillary column (Supelco-Sigma-Aldrich, St. Louis, MO, USA). FAME extracts (1 µL) were introduced through a split–splitless injection system of the autosampler in split mode (ratio 1:100) at 260 °C. The oven temperature program started at 140 °C (held for 5 min) and linearly increased to 260 °C (4 °C min^−1^) up to the end of the analysis. The FID temperature was 260 °C. The FA composition of samples was obtained by comparing with the retention time of the standard mixture FAME 37 components (Sigma-Aldrich). Relative abundance was obtained by software-assisted peak integration (Chemstation vers. B04.03, Agilent, Santa Clara, CA, USA) and was expressed as percentage area.

#### 2.2.3. Determination of Total Phenolic Content and Antioxidant Activity

The concentration of total phenols (TPC) in the hydroalcoholic extracts (80%, *v*/*v*, methanol) from GP, extra-virgin olive oil and biscuits was determined with the Folin–Ciocalteu colorimetric method, using the general procedures recommended by the European Pharmacopoeia for the determination of total tannins [19], monitoring the absorbance at 760 nm with a UV-Vis spectrophotometer (Amersham Ultrospec 2100 Pro UV/Vis, GE Healthcare, Uppsala, Sweden). Gallic acid within the 50–500 mg L^−1^ concentration range was used as the standard. TPC was expressed as g kg^−1^ of gallic acid equivalents (GAE). Samples were assayed in triplicate. The antioxidant capacity was determined as CDAC (coulometrically determined antioxidant capacity) according to a recently developed coulometric method [20].

#### 2.2.4. HPLC-DAD Analyses

The hydroalcoholic extracts (80% methanol) from GP, biscuits and the oral–gastro-duodenal digests of biscuits were separated using a modular HP 1100 chromatographer (Agilent Technologies, Palo Alto, CA, USA) equipped with a diode array detector (DAD). The stationary phase was a 250 × 2.0 mm i.d. C18 reversed-phase Jupiter column, 4 µm particle diameter (Phenomenex, Torrance, CA, USA), which was kept at 37 °C during the analyses. Runs were performed at a constant flow rate of 0.2 mL min^−1^ applying a 5–60% linear gradient of the solvent B (acetonitrile/0.1% TFA) after 5 min of isocratic elution at 5% B. Solvent A was 0.1% TFA in HPLC-grade water. For each analysis, 10 µL of combined extracts 10-fold diluted with 0.1% TFA were injected. Samples were run in triplicate, at least. DAD was set-up to acquire a UV-Vis spectrum every second between 200 and 600 nm. The HPLC separations were monitored at 520, 360, 320 and 280 nm.

### 2.3. In Vitro Oral–Gastro-Duodenal Digestion of Biscuit Prototypes

Simulated salivary fluid (SSF), simulated gastric fluid (SGF) and simulated intestinal fluid (SIF) were prepared according to the Infogest harmonized conditions [21,22]. One gram of individual ground biscuit samples, i.e., CTR, 20-GP, and 30-GP, were suspended in SSF (pH 7.0) including human salivary amylase (1500 U mL^−1^) and incubated for 2 min to mimic the oral phase. Subsequently, pH was adjusted at 3 with 1 M HCl, SGF including porcine pepsin (3300 U mg^−1^, final concentration of 12 mg mL^−1^) and phospholipids (10 mg mL^−1^) were incorporated and samples were incubated for 2 h at 37 °C. Pepsin hydrolysis was stopped by raising the pH to 7.0 with 1 M NaOH. To simulate the duodenal digestion, the gastric digest was two-fold diluted with SIF containing bile salts (10 mM in the final mixture, measured as cholic acid), and pancreatin from porcine pancreas (100 U mL^−1^ in the final mixture, based on the trypsin activity). The duodenal digestion was carried out for 2 h at 37 °C. Simulated chyle aliquots (0.5 mL) were sampled every 20 min during duodenal digestion to monitor the release of reducing sugars and phenolic compounds. Soon after sampling, 0.5 mL of pure methanol was included to stop the enzymatic activity. Hydroalcoholic mixtures were vigorously vortexed, centrifuged (5000× *g*, 20 min, 4 °C) and stored at 0 °C until analysis.

#### Determination of Reducing Sugars during Duodenal Digestion

Reducing sugars released during the duodenal phase of the digestion of biscuits were determined using a spectrophotometric assay based on the coupling reaction with 3,5-dinitrosalicylic acid (DNSA) as previously reported [23]. The DNSA solution (100 μL) was combined with 100 μL of the supernatants obtained from duodenal chyme at varying time points or with water for the blank and reacted in a boiling water bath for 15 min. After cooling on ice to room temperature, 900 μL deionized water was added, and the absorbance was read at 540 nm. A standard curve was built with 0.1–2 mg mL^−1^ of maltose prepared from a 0.2% stock solution in deionized water.

### 2.4. Determination of Volatile Organic Compound Profiles

Volatile organic compounds (VOCs) of biscuits were determined by HS-SPME/GC-MS. For sample preparation, 1.5 g of biscuits was individually weighed in a SPME 20 mL vial, added with 5 mL of saturated aqueous solution of NaCl and 10 µL of a solution of 3-octanol (Aldrich, Milan, Italy), used as internal standard (IS). Volatile components were measured according to Pasqualone et al. [24], with slight adaptations. Concisely, after equilibration at 40 °C for 30 min, extraction was performed by exposing the fiber (DVB-Carboxen-PDMS, 2 cm length SUPELCO, Bellefonte, PA, USA) in the HS of the vial at 40 °C for 50 min and then desorbed for 6 min at 240 °C in the injector port of the GC operating in splitless mode. An Agilent GC, model 7890A equipped with a mass spectrometer, model 5975 C (Agilent Technologies) was used. Volatile organic compounds (VOCs) were thermally released and separated by an HP-Innowax capillary column (30 m × 0.25 mm × 0.5 µm, Agilent J&W). The temperature of the oven was set at 40 °C for 5 min, increased to 230 °C at 5 °C min^−1^, then ramped to 260 °C at 15 °C min^−1^. The temperatures of the ion source and the quadrupole were set at 230 °C and 150 °C, respectively, and helium was used as the carrier gas. Mass spectra were recorded at 70 eV with the mass range scanned between 30 and 300 *m*/*z*. Each sample was measured in triplicate with blanks run every three analyses. Volatiles’ identification was carried out by using mass spectra comparisons to the available databases (NIST-2014/Wiley 7.0 libraries), by linear retention indices (LRI), measured by injecting C_8_-C_40_ *n*-alkane series (Supelco, Bellefonte, PA, USA) using the same chromatographic conditions and by commercial standards, when available. VOCs’ concentrations were quantified as the percent ratio of the individual compound peak area relative to the IS area (RPA%), and the volatile compounds’ areas were measured from the total ion chromatogram (TIC). 

### 2.5. Sensory Analysis

#### 2.5.1. Sample Preparation and Presentation

Freshly baked GP biscuits and the CTR biscuits were labeled with three-digit random numbers and served on a tray at room temperature (20 ± 2 °C). Sensory analysis was performed in individual booths equipped with tablets running a specific software for sensory data acquisition (FIZZ, Biosystemès, Couternon, France), according to the standard protocol UNI 8589 [25], at the CNR sensory lab in Bologna.

#### 2.5.2. Descriptive Analysis (DA)

Nine expert assessors with more than 70 h of training in sensory analysis, especially on baked products, performed the sensory descriptive analysis. Panelists were also previously trained in sensory descriptive methodology. Descriptive analysis tests performed by the expert panel were carried out in duplicate and samples were presented monadically in a balanced order. Panelists used water to rinse their mouths between samples. Before testing, panelists were informed of the main research outcomes and gave consent for their data to be used. Participation in the research was voluntary, and the right to privacy and data protection was respected following current legislation (GDPR 2016/679 [26]). Six sensory experts from the IBE sensory team compiled the terminology, rearranged the terms used in the literature [27,28] for other food or added news terms to create an appropriate lexicon list for the DA. Sensory attributes were used for the evaluation. Nine olfactory and aromatic attributes were chosen: dried fruits, red fruits, honey, raisin, citrus, peanut, toasted, spicy and off-odor/off-flavor. Moreover, five gustatory and eight texture attributes were also evaluated: sweet, acid, bitter, salty and umami; firmness, crunchiness, adhesiveness, gumminess, graininess, moisture, pungency and astringency. Finally, the panelists were also asked to rate the products’ overall liking. The descriptors were rated on a 9-point scale from “no perception” to the “highest intensity perceivable”.

### 2.6. Statistical Analysis

Analyses were performed in triplicate and the results were expressed as mean values followed by the standard deviation.

One-way analysis of variance (ANOVA), followed by the Tukey HSD test for multiple comparisons, was performed using the SPSS software package, Version 29.0 (SPSS Inc., Chicago, IL, USA), on the HS-SPME/GC-MS semi-quantitative data, to evaluate statistically significant differences among the volatile contents in each sample group.

For the sensory analysis, data were analyzed using IBM SPSS V. 29.0 and R programming language ver.4.3.1 (R Core Team 2023. _R: A Language and Environment for Statistical Computing_. R Foundation for Statistical Computing, Vienna, Austria) and SensoMineR: Sensory Data Analysis for R package version 1.2. One-way ANOVA analysis was performed on DA sensory scores, and the Tuckey post hoc test was carried out to test the differences between samples. The significance level was fixed at *p* < 0.05. Mean DA intensity values were used to generate a spider plot and represent the bars’ sensory profiles. A Principal Component Analysis (PCA) was performed on the matrix of the average scores of each sensory attribute, to summarize through a map the main characteristics of each bar sample.

## 3. Results and Discussion

**Figure 2:**
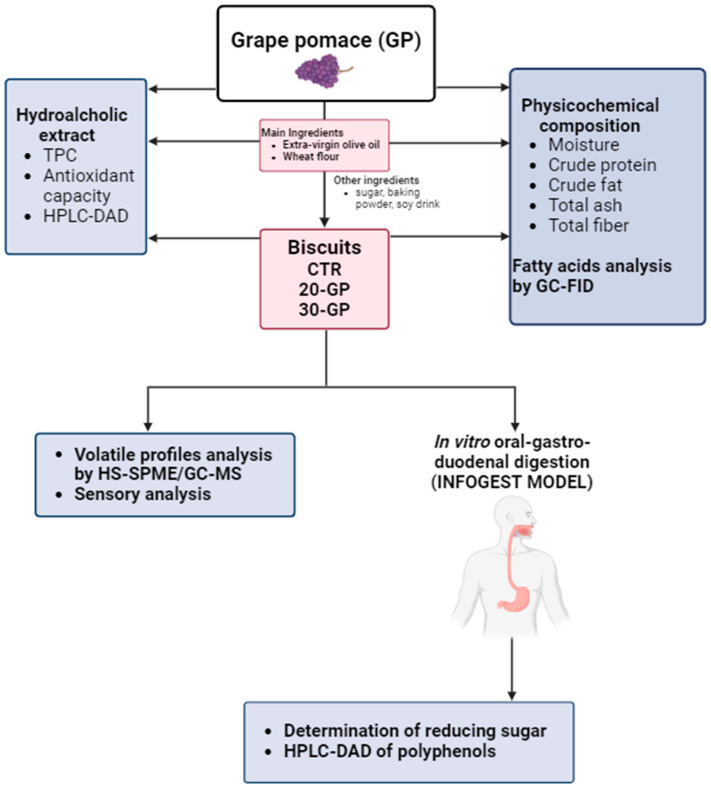
Schematic workflow of the experimental approach employed for the study and characterization of crude crushed grape pomace and their relative biscuit prototypes.

The characterization and the study of crude crushed red GP and their relative biscuit prototypes was determined using a multi-analytical approach (physicochemical composition, GC-FID, HPLC-DAD, HS-SPME/GC-MS and sensory analysis), according to the workflow diagram shown in Figure 2.

### 3.1. Characterization of Crude Crushed Grape Pomace and Biscuit Prototypes

#### 3.1.1. Proximate Composition, Energy Content and Fatty Acid Profile

Crude crushed GP was shown to be high in fiber (Table 1). As a consequence, biscuits with 20% and 30% GP contained 14.7% and 21.3% of fiber, compared to the 1.8% in the CTR biscuits. Conversely, lipids—mainly derived from EVO oil—were higher in the CTR formula (20.3%) compared to the 20 (15.2%) and 30 (14.7%) GP prototypes. As for the energy content, it was shown to decrease by around 50 kcal from the CTR biscuit to the 20% prototype, and by a further ~15 kcal when considering the 30 GP biscuit (Table 1). The fatty acid profile of GP is dominated by polyunsaturated fatty acids (PUFA, Table 2), especially because of grape seed-derived linoleic acid (61.68%), which is an essential fatty acid. Therefore, biscuits with 20 and 30% GP contained significantly higher amounts of linoleic acid than CTR ones. Similarly, GP increased the content of minerals as demonstrated by the determination of ash in biscuits (Table 2).

#### 3.1.2. Total and Specific Phenolic Content and Antioxidant Activity of Crude Crushed Grape Pomace and Biscuit Prototypes

The total polyphenol content (TPC) in crude crushed GP was 2495 ± 13 mg gallic acid equivalents per 100 g (Table 3). The TPCs in 20-GP and 30-GP biscuits were 456 ± 3 and 540 ± 2 mg GAE 100 g^−1^, respectively. As expected, the TPC was significantly higher in the 30-GP prototype compared to the 20-GP and the CTR biscuits (*p* < 0.05).

**Figure 3:**
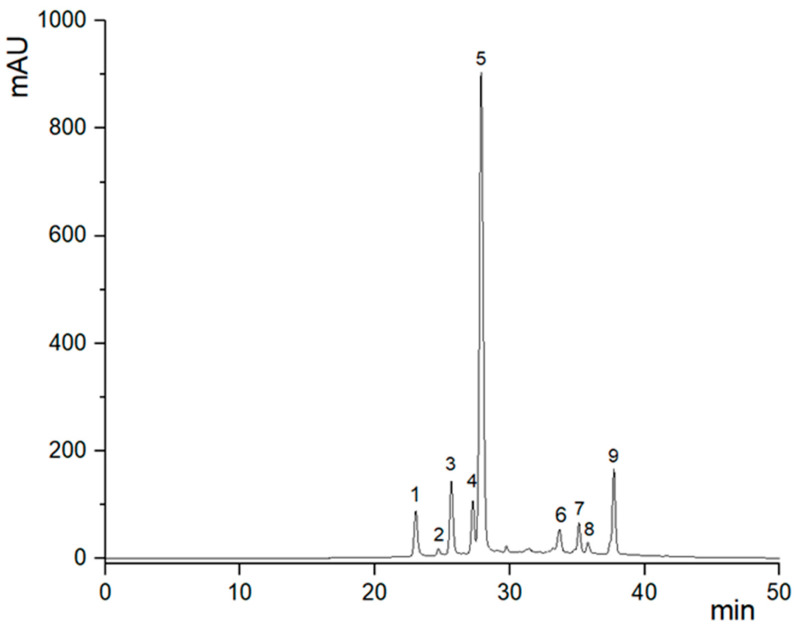
Representative HPLC chromatograms of a GP anthocyanins extract monitored at 520 nm. Peaks labelled in the figure are assigned from Table 4.

GP polyphenols exhibited the typical profile of *V. vinifera* grapes [29], with a particularly high content of anthocyanins dominated by oenin (malvidin 3-*O*-glucoside) as demonstrated by HPLC-UV (520 nm) analysis (Figure 3). Labelled peaks are assigned and quantified in Table 4. The content of anthocyanins in GP biscuits appeared unexpectedly low, likely due to their partial degradation during cooking, and the reduced extractability from the baked biscuits. GP also contains flavonoids and grape-seed procyanidins, detected by HPLC at 360 and 280 nm, respectively which largely contribute to the remarkable antioxidant capacity determined as CDAC (Table 3).

### 3.2. Oral–Gastro-Duodenal Digestion of Biscuits: Release of Reducing Sugars and Polyphenols

**Figure 4:**
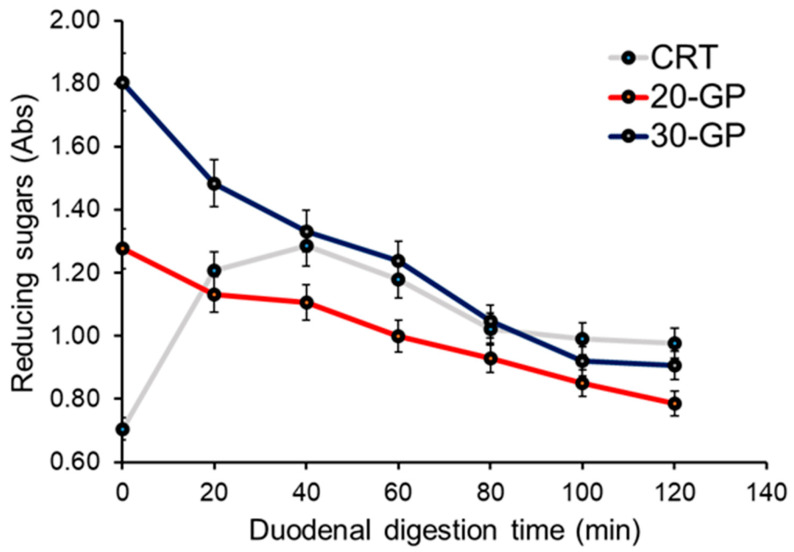
Release of reducing sugars from the starch during the duodenal digestion of control (CTR) biscuits (grey line) compared to 20-GP (red line) and 30-GP (blue line) biscuits.

**Figure 5:**
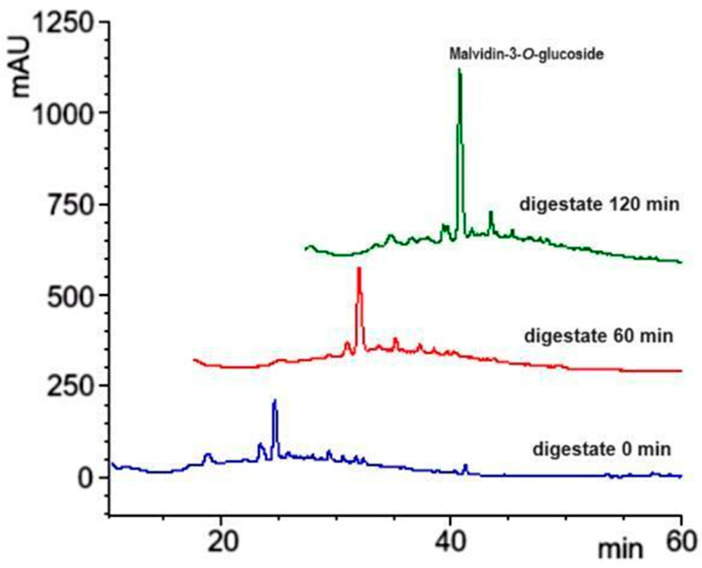
HPLC-DAD analysis (520 nm) of soluble extracts from 30-GP biscuits during simulated duodenal digestion demonstrating the progressively increasing release of anthocyanins.

During the oral–gastro-duodenal digestion of starch-based foods, reducing sugars are released by the effects of the action of salivary and, especially, pancreatic α-amylases. The release of reducing sugars was monitored during the duodenal phase of the simulated digestion of biscuits (Figure 4). As expected, in the CTR biscuits, the concentration of reducing sugars quickly increased during the early stages of duodenal digestion and reached a *plateau* after 1 h, showing a slightly decreasing trend afterwards. In the digests of 20-GP and 30-GP biscuits, the concentration of reducing sugars was higher than control biscuits at the beginning of the duodenal phase (time zero), likely due to the presence of reactive sugars introduced with the GP. Surprisingly, the concentration of reducing sugars for 20-GP and 30-GP biscuits dropped during the duodenal digestion, and the trend of decrease was consistent between the 20-GP and 30-GP biscuits. At longer duodenal digestion time points, all the samples tended to have a comparable concentration of reducing sugars. To exclude the interference of GP anthocyanins with the DNSA assay, considering that the coupling products of reducing sugars–DNSA are monitored at 540 nm that partly overlaps with the absorption bands of anthocyanins, the chyle samples were analyzed by HPLC. This analysis demonstrated that anthocyanins increased in the soluble fraction of chyle during digestion (Figure 5), due to the breakdown of the starch matrix induced by the concerted action of digestive enzymes. Therefore, the observed decrease in reducing sugars is not related to the amount of anthocyanins in the chyle.

Since the DNSA analysis accounts for reducing sugars in the supernatant of the digestion mixture, which is sampled during the duodenal digestion, the sugar decrease observed for the 20-GP and 30-GP biscuits might be attributed to non-covalent binding of sugars with progressively released methanol-insoluble fibers of GP, which subtract reducing sugars to the supernatant. Interestingly, the “sequestering” of sugars by fibers might have the physiological effects of diminishing the availability of simple saccharides for the intestinal uptake, thereby inducing a favorable reduction in the glycemic response.

Altogether, many compositional aspects suggest GP-enriched biscuits as possible functional foods with multiple health-promoting properties:

(i) A bulk of scientific evidence attributes antioxidative, anti-inflammatory and anti-hyperglycemic properties to polyphenols [30]. GP-derived anthocyanins and procyanidins are particularly effective as antioxidant compounds. After winemaking, approximately 70% of the grape polyphenols remain in the pomace [31]. Most of these compounds survive the cooking temperature and are then released during the digestion of GP-containing biscuits following the breakdown of the starch matrix and the dissolution of berry grape peels. (ii) GP is high in water soluble dietary fibers, which are desirable in foodstuff. Thus, the presence of GP significantly increases the fiber content of biscuits. GP fibers, especially those derived from red grape, have been classified as antioxidant fibers [32]. (iii) Dietary polyphenols exhibit inhibitory activity against human pancreatic α-amylase and intestinal α-glucosidase, in some instances comparable to acarbose, and can significantly reduce the postprandial glycemic response [33]. In particular, GP from red grape varieties has recently been identified as a source of compounds inhibiting the human enzymes responsible for the digestion of carbohydrates [34]. The combined action of GP fibers and polyphenols inhibiting α-amylase and α-glucosidase could significantly delay the absorption of glucose from starchy foods, supporting their potential for human use in the management of insulin resistance and type-2 diabetes [35].

### 3.3. Volatile Organic Compound Profiles

Overall, 60 VOCs were identified by HS-SPME/GC-MS analysis, including 12 aldehydes, 3 esters, 7 ketones, 6 terpenes, 7 alcohols, 5 pyrazines, 10 furans, 6 acids, 3 lactones and 1 pyrrole (Table 5 and Supplemental Table S2).

Most of the detected VOCs originated from the Maillard reaction, sugar caramelization or lipid peroxidation and are known to affect the overall aroma of biscuits [36].

Among the VOCs that play a central role in the final aroma of baked products, there are pyrazines (Py1–Py5), with a nutty, roasted and baked cereal smell, and furans (F1–F10), described as sweet, nutty, bready/almond- and caramel-flavored. Both of these chemical classes are formed by the Maillard reaction, which is the dominant chemical process responsible for the flavor formation during baking [14,24]. Volatile pyrazines are likely responsible for the biscuit flavor and biscuit odor associated to CTR biscuits from the sensory analysis (Table 6).

The incorporation of GP into the biscuits suppressed the formation of pyrazines, which were detected only in the CTR biscuits, while it increased the production of furan volatiles, which constituted almost 6, 35 and 39% of total VOCs in CTR, 20-GP and 30-GP biscuits, respectively (Table 5). A similar trend was found by Pasqualone et al. [24], who suggested that this result could be ascribed to the different pH values of the two types of biscuits. In particular, a pH lower than 7 seems to promote the formation of furan compounds, while higher pH values can favor the production of pyrazines [24]. On the other hand, the absence or the significant reduction in the pyrazine derivatives in GP biscuits compared to CTR biscuits has been explained by other authors with a possible inhibitory effect of phenolic compounds towards the formation of pyrazines, thanks to the ability of polyphenols to scavenge free radicals produced during the Maillard process [14,36,37].

The data in Table 5 show that esters, including ethyl acetate (E1), ethyl octanoate (E2) and ethyl decanoate (E3), were detected only in the pomace-enriched biscuits, consistent with previous studies in which esters were only identified in biscuits supplemented with winemaking by-products [14,24]. These findings can be explained with the fact that ester compounds are secondary or tertiary volatile constituents formed in wine through esterification, ester hydrolysis or primary or secondary fermentation [14]. Generally, the esters impart substantial floral and fruity notes to the wine aroma profile, in line with the sensory data, which associated the sensory parameters of wine odor and wine flavor principally to B20 and B30 biscuits (Table 6).

The results in Table 5 show a general increase in most aldehydes in GP biscuits compared to the CTR ones. Specifically, higher levels of benzaldehyde (Ald8) and benzenecetaldehyde (Ald9), which are VOCs with a high sensory impact, in GP-20 and GP-30 biscuits should stem from pomace by effect of the characteristic fermentative activities during the winemaking process [24].

Strecker’s aldehydes, including 2-methylpropanal (Ald1), 2-methylbutanal (Ald2) and 3-methylbutanal (Ald3), have been indicated as odor active compounds associated to malty notes in bakery products [38]. They were detected at higher levels in the GP-enriched biscuits than in CTR ones (Table 5), in contrast with Caponio et al. [14] who reported a decrease in these volatiles in the biscuits enriched with wine lees, associated with lower pH values. Conversely, our data are in line with Pasqualone et al. [24] who observed a positive correlation between Ald2 and Ald3 and the typical red and brown color tones of biscuits derived by the Maillard reaction.

Significant changes were also detected in the amount of lipid-derived VOCs, particularly saturated and α-unsaturated aldehydes and alcohols, which were more abundant in enriched than in model biscuits (Table 5). These changes could be imputable to the thermal oxidation of the unsaturated fatty acids which are abundant in the grape seeds of the pomace [24,39]. Providentially, the amounts of lipid-derived VOCs were far from generating rancid off-flavors in all tested biscuits, as supported by the sensory analysis (Table 6).

Finally, most of the observed carboxylic acids (A1-A6) resulting from wine fermentation processes were detected almost exclusively in the pomace-enriched biscuits (Table 5). These volatiles are generally considered responsible for the pungent notes [40].

Thus, as a general trend, the incorporation of pomace into the biscuits suppressed the formation of volatile pyrazines while enhancing the production of furans and the release of VOCs resulting from grape fermentation as well as the autoxidation of the unsaturated lipids of grape seeds. The lower content of volatile compounds (i.e., pyrazine derivatives) formed by the Maillard reactions in GP biscuits than control biscuits possibly depends on the ability of polyphenols to act as free radical scavengers. This suggests that GP polyphenols could mitigate the formation of advanced glycation end products, which are increasingly proposed as one major link between unhealthy diets and inflammation, type 2 diabetes and obesity [41,42].

### 3.4. Sensory Properties

Sensory data obtained through descriptive analysis (DA) highlighted clear statistical differences between the CTR sample and the GP biscuit prototypes (Table 6). The differences among the GP biscuits were mainly related to texture attributes.

Indeed, the GP biscuits were characterized by higher winy and red fruits aromatic and olfactory notes, while the CTR sample was higher in EVO notes. The latter was also described by a more pronounced biscuit flavor and sweetness, while GP biscuits were higher in bitterness and acidity.

In relation to the mouthfeel sensations, the CTR sample was higher in greasiness but also in crunchiness and friability, which both characterized the 20-GP biscuit as well. The 30-GP sample was the most chewy, firm and astringent but also seedy, together with the 20-GP biscuits.

Concerning the judges’ hedonic appreciation, the most liked sample was the CTR, followed by the 20-GP biscuit.

DA results obtained through the panel test were also analyzed in a multidimensional way through PCA, which showed that the first and the second principal components accounted for 81.1% and 18.9%, respectively, of the experimental variability in the data, representing 100% (Supplemental Figure S1). The three samples were clearly separated in three different quarters and characterized by different attributes.

The first dimension (81.1% of variance), representing the most variability, was positively correlated with the main attributes related to GP biscuits: winy, red dried fruits notes, bitter and acid, and mouthfeel sensations as seedy, firmness, chewiness, adhesiveness astringent and pungent. Moreover, the first dimension was negatively correlated with attributes that clearly characterized the control sample: EVO oil notes, biscuit flavor and sweet, and texture attributes as greasiness, crunchiness and friability, and the overall liking.

The second dimension (18.9% of variance) was positively correlated with the attribute graininess, mainly related to the 20-GP biscuit, and negatively correlated with salty, mainly related to the 30-GP biscuit.

## 4. Conclusions

In this study, we demonstrated that a simple plant-based biscuit formula can be successfully enriched with whole crushed grape pomace up to a high rate (30%), to obtain prototypes with improved nutritional properties able to increase the habitual intake of polyphenols. These bioactive compounds have been associated with a risk reduction for cardiometabolic and cardiovascular disease incidence by epidemiological evidence. The lower availability of simple sugars for the intestinal absorption observed in vitro for GP biscuits compared to the control formula is likely to be the result of a combined action of GP fibers and polyphenols. Moreover, the presence of polyphenols in GP biscuits appears to mitigate the formation of advanced glycated end products.

In spite of the high percentages of GP utilized, the sensorial attributes and the overall liking of the products developed—especially those with 20% of GP—were not substantially affected, as compared to the base formula.

Overall, the data here provided would support clinical studies assessing the potential role as functional foods of the GP biscuits, and, afterwards, the large-scale production of a food mainly based on a by-product ingredient, with a high nutritional value and hedonistically appreciated, potentially able to deliver benefits for the cardiometabolic health at the population level. 

## Figures and Tables

**Table 1:** Chemical and nutritional properties of crude crushed grape pomace (GP) and biscuits with different rates of GP as well as the control (CTR) biscuit. The variability, evaluated as relative standard deviation, was lower than 5% in all the cases and it is not reported.

Product	Moisture (%)	Ash (%)	Protein (%)	Total Lipid (%)	Total Carbohydrates (%)	Dietary Fiber (%)	Available Carbohydrates (%)	Energy (kcal 100 g^−1^)
GP	14.8	3.2	6.0	6.1	70.0	55.0	15.0	245.2
CTR	5.7	1.2	7.3	20.3	65.5	1.8	63.7	454.4
20-GP	4.5	2.1	5.8	15.2	72.4	14.7	57.7	405.8
30-GP	4.6	2.0	4.8	14.7	74.0	21.3	52.7	391.7

**Table 2:** Fatty acid composition of crude crushed grape pomace (GP) and biscuit prototypes.

Fatty Acids (% Area)	GP	CTR	20-GP	30-GP
Palmitic, C16:0	7.69 ± 0.22	14.10 ± 0.12 ^a^	14.7 ± 0.02 ^b^	14.61 ± 0.04 ^b^
Palmitoleic, C16:1	0.21 ± 0.01	0.72 ± 0.01 ^a^	0.75 ± 0.04 ^a^	0.63 ± 0.03 ^b^
Stearic, C18:0	5.28 ± 0.35	1.50 ± 0.03 ^a^	1.84 ± 0.02 ^b^	2.07 ± 0.03 ^c^
Oleic, C18:1 ω-9 *c*	21.52 ± 0.35	56.36 ± 0.10 ^a^	54.65 ± 0.04 ^b^	50.6 ± 0.02 ^c^
Linoleic, C18:2 ω-6 *c*	61.68 ± 0.35	22.33 ± 0.22 ^a^	23.59 ± 0.03 ^b^	25.93 ± 0.03 ^c^
Arachidic, C20:0	0.53 ± 0.25	0.15 ± 0.01 ^a^	0.25 ± 0.01 ^b^	0.32 ± 0.02 ^c^
Linolenic, C18:3 ω-3	0.9 ± 0.01	1.56 ± 0.02 ^a^	2.00 ± 0.02 ^b^	2.26 ± 0.02 ^c^
*cis*-11-eicosenoico, C20:1	0.23 ± 0.15	0.20 ± 0.03 ^a^	0.22 ± 0.01 ^a^	0.24 ± 0.01 ^a^
Σ-SFA	13.50 ± 0.28	15.75 ± 0.10 ^a^	16.79 ± 0.02 ^b^	17.00 ± 0.05 ^c^
Σ-MUFA	22.64 ± 0.13	60.28 ± 0.05 ^a^	57.43 ± 0.06 ^b^	54.01 ± 0.12 ^c^
Σ-PUFA	62.58 ± 0.36	23.89 ± 0.21 ^a^	25.59 ± 0.03 ^b^	28.19 ± 0.01 ^c^
Σ-PUFA/Σ-SFA	4.63 ± 0.70	1.52 ± 0.01 ^a^	1.52 ± 0.01 ^a^	1.66 ± 0.01 ^b^
ω-6/ω-3	68.80 ± 0.17	14.30 ± 0.26 ^a^	11.77 ± 0.06 ^b^	11.50 ± 0.10 ^b^

For each fatty acid in the biscuit prototypes, the mean values followed by different letters (^a,b,c^) are significantly different (*p* ≤ 0.05) according to Bonferroni post hoc test.

**Table 3:** Total polyphenol content and antioxidant activity (CDAC) of crushed GP and biscuit prototypes.

	GP	CTR	20-GP	30-GP
Total polyphenols (mg_GAE_ 100 g^−1^)	2495 ± 13	30 ± 1 ^a^	456 ± 3 ^b^	540 ± 2 ^c^
CDAC (mmol e^−^ 100 g^−1^)	125 ± 3	4 ± 1 ^a^	17 ± 1 ^b^	19 ± 1 ^c^

The mean values of total polyphenols and CDAC in the biscuit prototypes followed by different letters (^a,b,c^) are significantly different (*p* ≤ 0.05) according to Bonferroni post hoc test.

**Table 4:** Anthocyanin content in the crashed crude GP and in the biscuit prototypes. The variability, evaluated as relative standard deviation, was lower than 5% in all the cases (not reported).

N.	Compound (mg kg^−1^)	GP	CTR	20-GP	30-GP
1	Delphinidin-3-*O*-glucoside	291.3	-	0.4	1.2
2	Cyanidin-3-*O*-glucoside	55.1	-	0.2	0.3
3	Petunidin-3-*O*-glucoside	618.9	-	1.2	2.9
4	Peonidin-3-*O*-glucoside	404.7	-	0.9	2.1
5	Malvidin-3-*O*-glucoside	4657.0	-	9.6	22.8
6	Malvidin-3-*O*-(6-*O*-acetyl)glucoside	266.7	-	0.3	0.9
7	Delphinidin-3-*O*-(6-*O*-*p*-coumaroyl)glucoside	245.5	-	0.4	2.1
8	Peonidin-3-*O*-(6-*O*-*p*-coumaroyl)glucoside	105.4	-	0.3	0.6
9	Malvidin-3-*O*-(6-*O*-*p*-coumaroyl)glucoside	681.2	-	4.2	9.1

**Table 5:** One-way ANOVA performed on the HS-SPME/GC-MS data detected in control (CTR) biscuits and biscuits at different rates of GP (20-GP and 30-GP).

Volatiles	Code	CTR	20-GP	30-GP
2-Methypropanal *	Ald1	8.3 ^a^	10.3 ^b^	16.6 ^c^
2-Methylbutanal *	Ald2	21.8 ^a^	24.4 ^b^	31.6 ^c^
3-Methylbutanal **	Ald3	87.4 ^a^	126.5 ^b^	197.0 ^c^
Hexanal *	Ald4	9.7 ^a^	14.9 ^b^	20.1 ^c^
Octanal **	Ald5	0.0 ^a^	14.3 ^c^	13.5 ^b^
*trans*-2-Heptenal **	Ald6	0.0 ^a^	0.0 ^a^	18.1 ^b^
Nonanal **	Ald7	80.0 ^b^	48.5 ^a^	57.8 ^ab^
*trans*-2-Octenal ***	Ald8	0.0 ^a^	18.9 ^c^	14.3 ^b^
Benzaldehyde ***	Ald9	19.5 ^a^	22.6 ^b^	31.5 ^c^
Benzeneacetaldehyde **	Ald10	26.0 ^a^	69.2 ^b^	78.4 ^c^
2-Nonenal ***	Ald11	0.0 ^a^	0.0 ^a^	28.6 ^b^
*trans-trans*-2,4-Decadienal **	Ald12	0.0 ^a^	22.6 ^c^	13.6 ^b^
Ethyl acetate **	E1	0.0 ^a^	0.4 ^b^	1.2 ^c^
Ethyl octanoate **	E2	0.0 ^a^	7.2 ^b^	15.2 ^b^
Ethyl decanoate **	E3	0.0 ^a^	3.2 ^b^	6.2 ^c^
2,3-Butanedione **	K1	14.4 ^ab^	24.7 ^b^	9.7 ^a^
2,3-Pentanedione **	K2	15.2 ^ab^	18.8 ^b^	7.5 ^a^
3-Octanone	K3	16.6	25.0	21.5
1-Octen-3-one **	K4	0.0 ^a^	0.0 ^a^	9.6 ^b^
2-Propanone, 1-hydroxy-(Acetol) **	K5	21.6 ^a^	71.5 ^b^	14.0 ^a^
Acetoin **	K6	0.0 ^a^	13.5 ^b^	13.5 ^b^
6-Methyl-5-heptene-2-one ***	K7	0.0 ^a^	0.0 ^a^	7.9 ^b^
α-Pinene *	T1	41.1 ^b^	18.3 ^a^	18.3 ^a^
α-Phellandrene *	T2	40.8 ^b^	14.3 ^a^	14.3 ^a^
β-Pinene *	T3	47.9 ^b^	11.1 ^a^	11.1 ^a^
Limonene **	T4	521.5 ^c^	25.3 ^a^	236.6 ^b^
γ-Terpinene *	T5	71.3 ^a^	23.9 ^b^	23.9 ^b^
*o*-Cymene *	T6	17.5 ^b^	4.9 ^a^	4.9 ^a^
1-Hexanol ***	Alc1	3.3 ^a^	13.3 ^c^	10.5 ^b^
1-Octen-3-ol **	Alc2	0.0 ^a^	5.6 ^b^	6.6 ^b^
2-Ethyl-1-hexanol **	Alc3	0.0 ^a^	0.0 ^a^	12.9 ^b^
1-Octanol	Alc4	8.7	12.5	12.5
Benzenmethanol **	Alc5	3.8 ^a^	9.8 ^b^	8.6 ^b^
Benzeneethanol *	Alc6	6.5 ^a^	13.5 ^b^	14.2 ^b^
4-Vinyl-2-methoxy-phenol **	Alc7	0.0 ^a^	3.1 ^b^	6.6 ^ab^
Pyrazine, methyl **	Py1	38.6 ^b^	0.0 ^a^	0.0 ^a^
2,5-Dimethylpyrazine **	Py2	11.1 ^b^	0.0 ^a^	0.0 ^a^
2-Ethylpyrazine **	Py3	57.2 ^b^	0.0 ^a^	0.0 ^a^
Pyrazine, 2,3-dimethyl **	Py4	9.4 ^b^	0.0 ^a^	0.0 ^a^
2-Ethyl-6-methylpyrazine **	Py5	13.3 ^b^	0.0 ^a^	0.0 ^a^
2-Pentylfuran *	F1	15.4 ^a^	9.7 ^b^	9.2 ^b^
Dihydro-2-methyl-3(2H)-furanone **	F2	0.0 ^a^	6.9 ^b^	6.9 ^b^
2-Furancarboxaldehyde; Furfural **	F3	23.6 ^a^	472.2 ^b^	486.6 ^b^
Ethanone, 1-(2-furanyl); 2-Acetylfuran **	F4	0.0 ^a^	26.9 ^b^	33.5 ^b^
5-Methylfurfural *	F5	0.0 ^a^	30.7 ^b^	30.7 ^b^
5-Hydroxymethylfurfural ***	F6	0.0 ^a^	20.2 ^c^	6.5 ^b^
2-Furanmethanol **	F7	33.6 ^a^	158.7 ^b^	104.1 ^b^
5-Methyl-2-furfurylalcohol ***	F8	0.0 ^a^	48.4 ^c^	29.7 ^b^
2(5H)-Furanone	F9	8.1	8.4	8.4
4-Hydroxy-2,5-dimethylfuran-2(3H)-one (Furaneol) **	F10	0.0 ^a^	6.0 ^b^	2.8 ^ab^
Acetic acid ***	A1	15.3 ^a^	457.7 ^c^	224.8 ^b^
Propanoic acid **	A2	0.0 ^a^	15.8 ^b^	13.4 ^b^
Butanoic acid **	A3	0.0 ^a^	13.1 ^b^	10.8 ^b^
Pentanoic acid **	A4	0.0 ^a^	6.4 ^b^	7.6 ^b^
Hexanoic acid ***	A5	2.7 ^a^	37.9 ^b^	30.0 ^b^
Octanoic acid ***	A6	0.0 ^a^	3.1 ^c^	1.8 ^b^
Butyrolactone **	L1	0.0 ^a^	7.0 ^b^	7.0 ^b^
2-Hydroxy-γ-butyrolactone **	L2	0.0 ^a^	11.9 ^b^	3.1 ^a^
3,5-Dihydroxy-6-methyl-2,3-dihydro-4H-pyran-4-one ***	L3	0.0 ^a^	6.1 ^b^	4.7 ^b^
Ethanone, 1-(1H-pyrrol-2-yl); 2-Acetylpyrrole **	Pyr1	1.7 ^a^	11.5 ^b^	10.0 ^b^

For each parameter, the mean values followed by different letters (^a,b,c^) are significantly different (*p* ≤ 0.05) according to Tukey post hoc test (* *p* ≤ 0.05; ** *p* ≤ 0.001; *** *p* ≤ 0.0001).

**Table 6:** Mean intensity scores for the sensory attributes of the different biscuits.

Attribute	CTR	20-GP	30-GP
Winy odor ***	1.4 ^b^	2.8 ^a^	2.7 ^a^
Dried red fruit odor ***	1.3 ^b^	2.9 ^a^	3.1 ^a^
EVO oil odor *	4.6 ^a^	4.1 ^ab^	4.0 ^b^
Biscuit odor	3.8	3.5	3.6
Sweet ***	5.2 ^a^	4.2 ^b^	4.2 ^b^
Salty	2.7	2.7	2.9
Bitter ***	1.7 ^b^	2.9 ^a^	2.7 ^a^
Acid **	1.7 ^b^	2.4 ^a^	2.7 ^a^
Winy flavor ***	1.5 ^b^	3.5 ^a^	4.0 ^a^
Dried red fruit flavor ***	1.6 ^b^	3.8 ^a^	4.1 ^a^
EVO oil flavor	3.9	3.8	3.8
Biscuit flavor ***	4.9 ^a^	3.9 ^b^	3.6 ^b^
Firmness ***	4.8 ^c^	5.9 ^b^	6.6 ^a^
Crunchiness ***	6.0 ^a^	6.3 ^a^	5.0 ^b^
Friability ***	6.2 ^a^	5.7 ^a^	3.7 ^b^
Chewiness ***	3.5 ^c^	4.7 ^b^	5.9 ^a^
Greasiness ***	3.9 ^a^	3.1 ^b^	2.9 ^b^
Graininess	5.8	6.2	5.6
Adhesiveness	3.5	3.7	3.9
Seedy ***	1.4 ^b^	5.7 ^a^	5.8 ^a^
Pungent	2.3	2.6	2.8
Astringent **	3.2 ^b^	3.6 ^ab^	4.1 ^a^
Liking *	6.1 ^a^	5.9 ^ab^	5.2 ^b^

Different letters (^a,b,c^) correspond to different mean values according to one-way Anova followed by Tuckey post hoc test (* *p* < 0.05; ** *p* < 0.01; *** *p* < 0.001).

## Data Availability

The original contributions presented in the study are included in the article/Supplementary Material, further inquiries can be directed to the corresponding authors.

## Supplementary Materials

The following supporting information can be downloaded at: https://www.mdpi.com/article/10.3390/foods13142195/s1, Table S1. Wire-cut biscuit formulas. Table S2. Volatile compounds, their code, the linear retention times (Calculated vs. Literature) and the identification methods. Figure S1. Representation of the samples and the sensory attributes in the first two dimensions of the PCA analysis using descriptive analysis data.
