# Supplementary material for: Turning Wastes into Resources: Red Grape Pomace-Enriched Biscuits with Potential Health-Promoting Properties
Source: Foods. 2024 Jul 11;13(14):2195. doi: 10.3390/foods13142195 (PMC11276511; doi:10.3390/foods13142195)

Supplementary Material

Supplemental Table S1. Wire-cut biscuit formulas.

Ingredient	CTR biscuits (g)	20-GP biscuits (g)	30-GP biscuits (g)
Zero organic wheat flour	40	32	28
White sugar	20	16	14
Extra-virgin olive oil	20	16	14
Organic soy drink with no added sugars	15	12	10.5
Organic baking powder	5	4	3.5
Whole grape pomace flour	0	20	30

Supplemental Table S2. Volatile compounds, their code, the linear retention times (Calculated vs Literature) and the identification methods

Chemical class	Compound identity	Code	LRI^{Calc} / LRI^{Ref}	ID Method
Alcohol	Ethanol	Alc1	935/935	RI/MS
	2-Methyl-1-propanol	Alc2	1083/1078	RI/MS
	2-Pentanol	Alc3	1111/119	RI/MS/S
	3-methyl-1-butanol	Alc4	1201/1200	RI/MS
	1-Pentanol	Alc5	1244/1251	RI/MS/S
	2-Heptanol	Alc6	1310/1321	RI/MS
	1-Hexanol	Alc7	1340/1339	RI/MS/S
	2-Nonanol	Alc8	1510/1522	RI/MS
	Phenylethyl alcohol	Alc9	1918/1920	RI/MS/S
Aldehyde	2-Methylbutanal	Ald1	872/880	RI/MS/S
	3-Methylbutanal	Ald2	899/899	RI/MS/S

Acids	Acetic acid	A1	1460/1460	RI/MS/S
	2-methylpropanoic acid	**A2**	1573/1573	RI/MS/S
	Butyric acid	**A3**	1631/1631	RI/MS/S
	3-Methylbutanoic acid	**A4**	1689/1686	RI/MS
	Pentanoic acid	**A5**	1730/1733	RI/MS/S
	Hexanoic acid	A6	1855/1854	RI/MS/S
	Heptanoic acid	A7	1962/1960	RI/MS/S
	Octanoic acid	A8	2066/2067	RI/MS/S
	Nonanoic acid	A9	2169/2169	RI/MS/S
	Decanoic acid	A10	2261/2261	RI/MS/S
Ester	Ethyl acetate	E1	869/870	RI/MS/S
	Ethyl butyrate	E2	989/989	RI/MS/S
	Isoamyl butyrate	E3	1266/1267	RI/MS/S
	Isoamyl acetate	E4	1121/1122	RI/MS
	Ethyl pentanoate	E5	1138/1140	RI/MS/S
	Isobutyl butyrate	E6	1158/1162	RI/MS
	Pentyl acetate	E7	1170/1172	RI/MS
	Isopropyl hexanoate	E8	1228/1228	RI/MS/S
	Ethyl hexanoate	E9	1252/1251	RI/MS/S
	Isoamyl butyrate	E10	1277/1267	RI/MS/S
	Ethyl-3-hexenoate	E11	1294/1291	RI/MS
	Propyl hexenoate	E12	1324/1224	RI/MS
	Ethyl heptanoate	E13	1331/1331	RI/MS/S
	Heptyl acetate	E14	1385/1384	RI/MS
	Isobutyl hexanoate	E15	1399/1400	RI/MS
	Ethyl octanoate	E16	1445/1446	RI/MS/S
	Ethyl octanoate	E17	1448/1448	RI/MS/S
	Isoamyl hexanoate	E18	1450/1453	RI/MS

	Methyl decanoate	E19	1596/1593	RI/MS/S
	Ethyl decanoate	E20	1644/1642	
Ketones	Acetone	K1	< 800	RI/MS
	2-Butanone	K2	926/924	MS/LRI/S
	2-Pentanone	K3	981/981	RI/MS
	2-Hexanone	K4	1082/1083	RI/MS
	2-Heptanone	K5	1190/1193	RI/MS/S
	2-Octanone	K6	1418/1419	RI/MS
	Acetoin	K7	1316/1314	RI/MS
	2-Nonanone	K8	1386/1387	RI/MS/S
	8-Nonen-2-one	K9	1418/1421	RI/MS
	2-Decanone	K10	1688/1692	RI/MS
	2-Undecanone	K11	1603/1600	RI/MS/S
Others	γ-Caprolactone	O1	1708/1708	RI/MS
	β-Pinene	O2	1106/1118	RI/MS/S
	Limonene	O3	1214/1215	RI/MS/S

LRI_t: Relative retention indices on polar column reported in literature by www.pherobase.com; www. flavornet.org; www.ChemSpider.com; webbook.nist.gov; LRI_{sp}: Linear retention indices calculated against n-alkanes (C_8-C_{40}) on HP-Innowax column; Identification method as indicated by the following: LRI: Linear retention index on a on HP-Innowax column; MS: NIST and Wiley libraries spectra; S: co-injection with authentic standard compounds on the HP-Innowax column.

Supplemental Figure S1. Representation of the samples and the sensory attributes in the first two dimensions of the PCA analysis using descriptive analysis data.

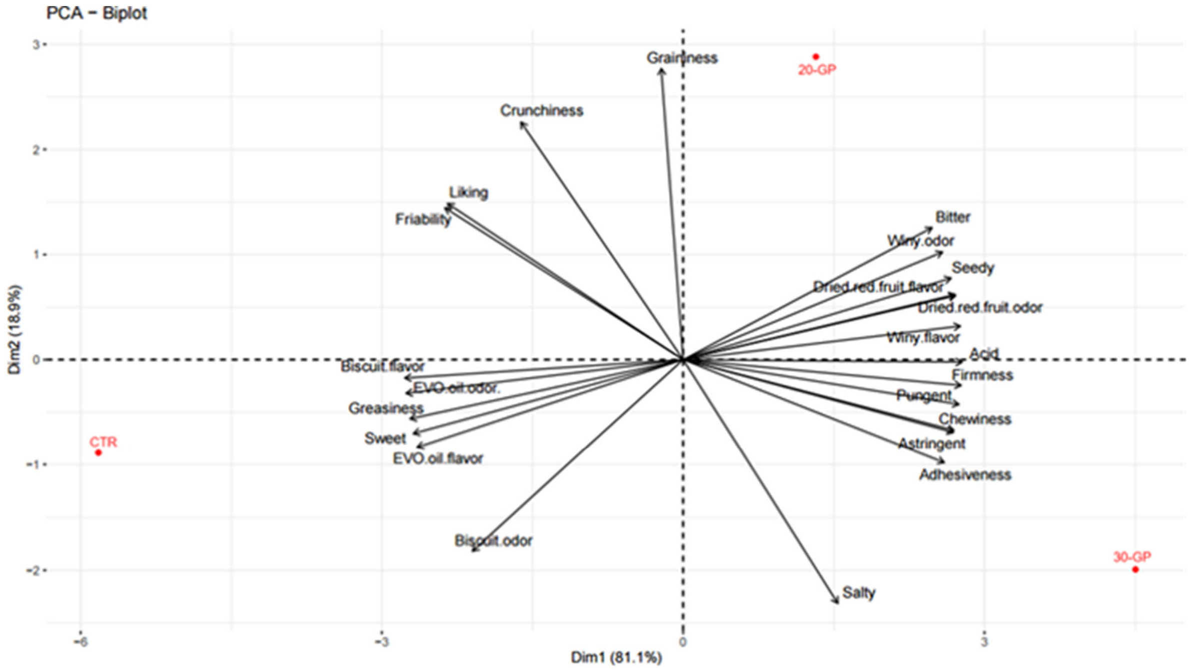

Supplement: Supplementary file 1 [file foods-13-02195-s001.zip › foods-3087145-supplementary.pdf]
